# Supplementary material for: Enhancing neuronal chloride extrusion rescues α2/α3 GABAA-mediated analgesia in neuropathic pain
Source: Nat Commun. 2020 Feb 13;11:869. doi: 10.1038/s41467-019-14154-6 (PMC7018745; doi:10.1038/s41467-019-14154-6)
Supplement: Supplementary file 2 — Supplementary Information [file 41467_2019_14154_MOESM2_ESM.pdf]

Supplementary Information

Enhancing neuronal chloride extrusion rescues α_2/α_3GABA$_A$-mediated analgesia in neuropathic pain

Lorenzo et al.

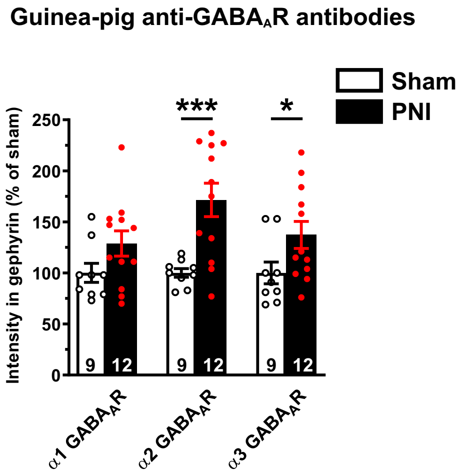

Supplementary figure 1: Quantification with anti-α-GABAAR antibodies raised in another species. Synaptic immunostaining intensities for the α1,2,3-subunits, for sham and PNI rats, using anti-$GABA_A$R antibodies raised in guinea pig and provided by Dr Jean-Marc Fritschy Laboratory. Results obtained with these antibodies were similar to the ones obtained using the *Synaptic System* antibodies (Fig. 2c) even if they recognize different epitope. (*P < 0.05; ***P < 0.001).

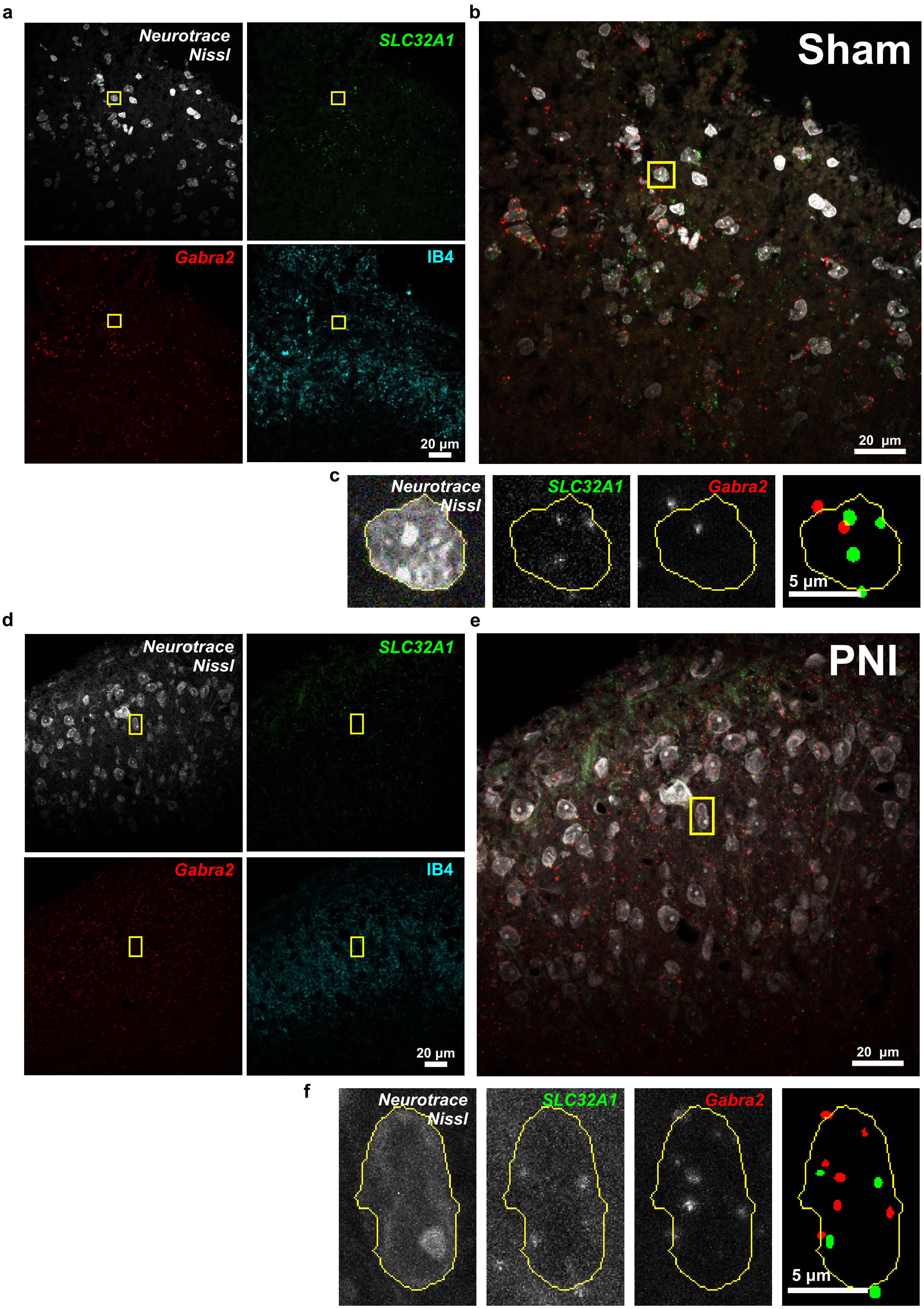

Supplementary figure 2: *Gabra2* over-expression in *SLC32A1*-positive inhibitory neurons.
a Confocal images of Neurotrace Nissl staining coupled with RNAscope multiplex staining in the dorsal horn of sham rats. The *SLC32A1* probe was used to reveal VGAT-positive neurons among the Nissl-positive population. The number of *Gabra2* mRNA clusters in VGAT-positive neurons were quantified and averaged within the IB4-positive band to define a *Gabra2* mRNA copy index. **b** Merged confocal image of the four images in **a**. **c** Enlargement of the inset in **b** showing the respective labeling at higher magnification. **d, e, f** same as **a, b, c** but in PNI rats.

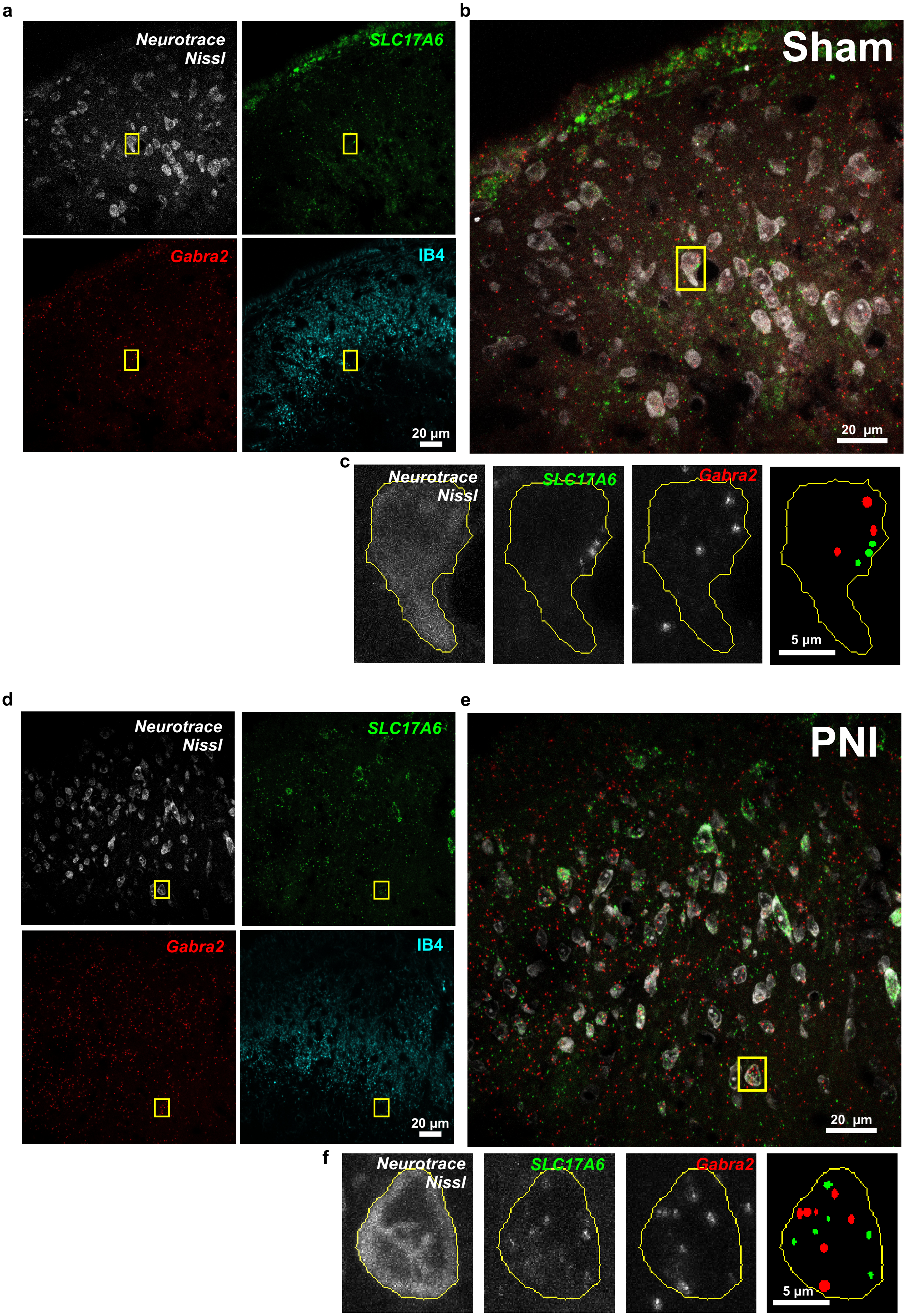

Supplementary figure 3: *Gabra2* over-expression in *SLC17A6*-positive excitatory neurons.
a Confocal images of Neurotrace Nissl staining coupled with RNAscope multiplex staining in the dorsal horn of sham rats. The *SLC17A6* probe was used to reveal VGluT2-positive neurons among the Nissl-positive population. The number of *Gabra2* mRNA clusters in VGluT2-positive neurons were quantified and averaged within the IB4-positive band to define a *Gabra2* mRNA copy index. **b** Merged confocal image of the four images in **a**. **c** Enlargement of the inset in **b** showing the respective labeling at higher magnification. **d, e, f** same as **a, b, c** but in PNI rats.

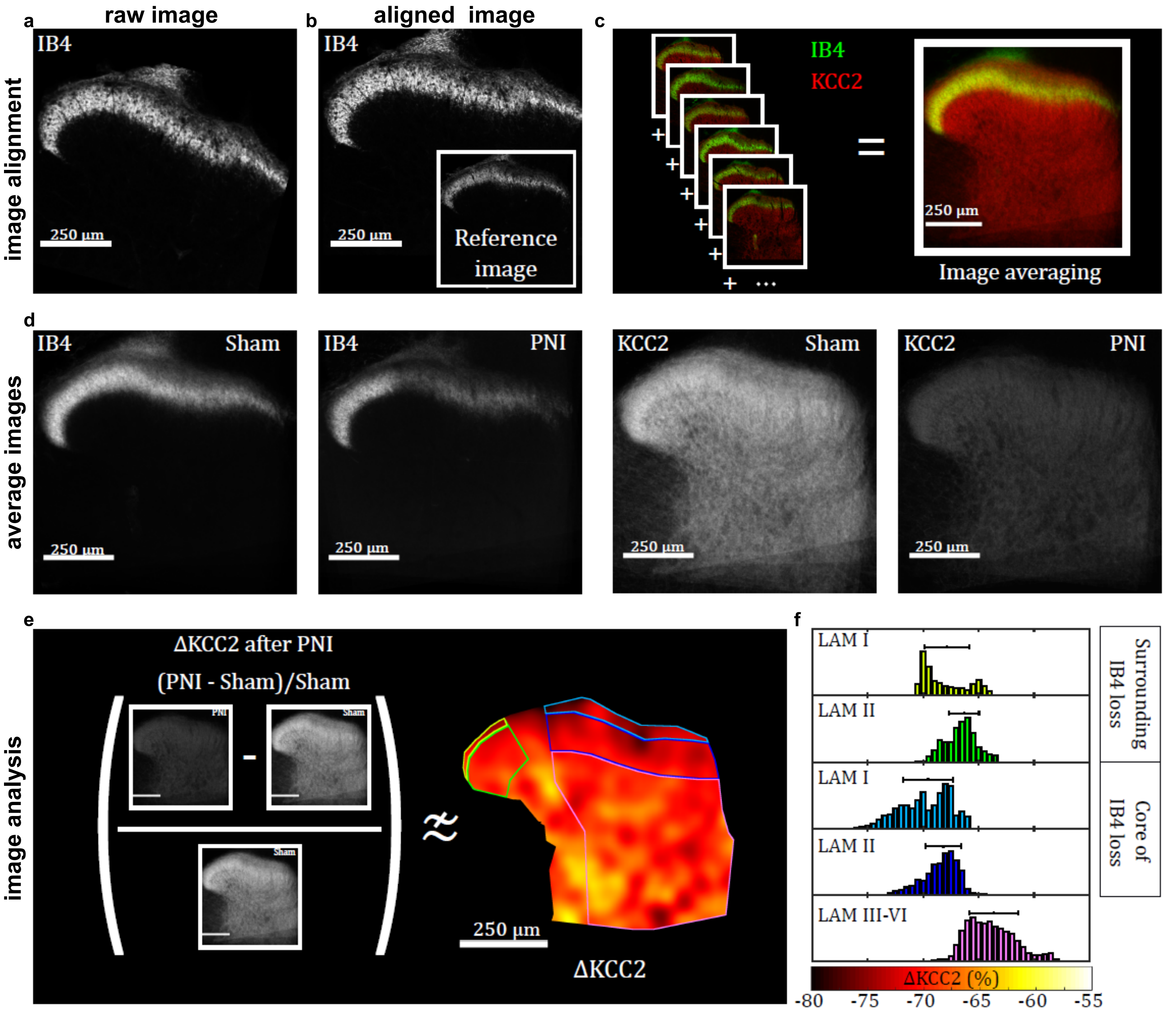

Supplementary figure 4: Mapping of the KCC2 loss in the rat spinal cord dorsal horn after PNI.
a Raw confocal tile images (11 x 11 images, objective 20 zoom 5, see methodological section) of the IB4 staining observed in the rat spinal dorsal horn. **b** Aligned IB4 image (rotation + translation) to overlap the selected reference IB4 image. **c.** Method of pixel per pixel averaging of IB4 and KCC2 transformed images. **d** Averages of all the aligned IB4 and KCC2 images in sham and PNI rats. **e** Relative difference in KCC2 pixel intensity between shams and PNIs in the dorsal horn and selection of the subregions at different depth from the white matter and at different distances of the IB4 loss core **f** Histograms of the pixel distribution (number of pixels in y-axis) in function of the variation of KCC2 average pixel immunostaining intensity (ΔKCC2 in x-axis) between shams and PNIs for each selected subregions of the rat spinal dorsal horn.

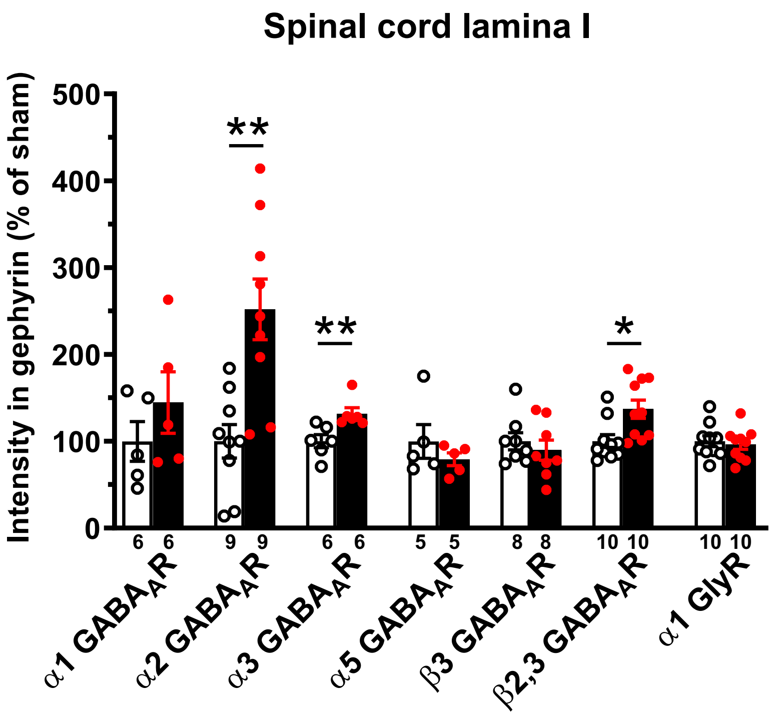

Supplementary figure 5: Postsynaptic scaling and $GABA_AR$ subunit switch in lamina I after peripheral nerve injury (PNI).

Identical analysis as in Fig. 2c but in lamina I of the rat spinal dorsal horn, instead of lamina II (region of loss of IB4 afferent terminals Fig. 2).

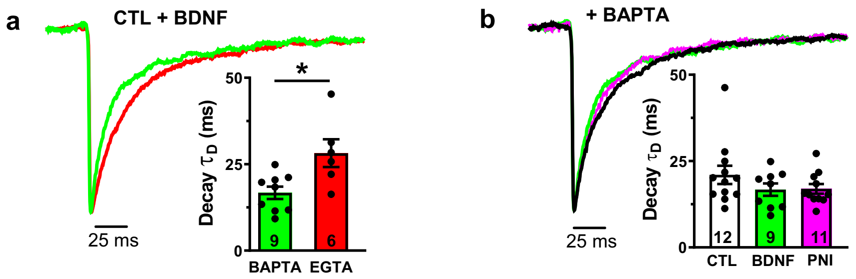

Supplementary figure 6: Kinetics of the GABAergic mIPSCs depend on the Ca^{2+} chelator type used for recording.

a $GABA_A$ mIPSC decays in BDNF conditions were significantly faster in BAPTA as compared to EGTA. **b** With BAPTA in the internal pipette solution, no significant difference in the $GABA_A$ mIPSC decays could be seen between control, BDNF and PNI conditions. (*$P < 0.05$).

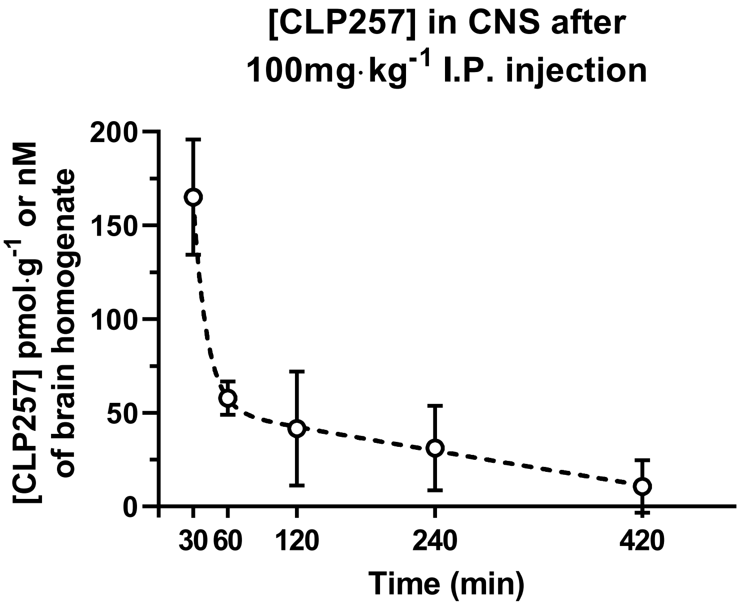

Supplementary figure 7: CNS penetration and clearance of CLP257 after i.p. injection.

Concentrations of CLP257 were measured by LC/MS (Liquid Chromatography / Mass Spectrometry) from brain homogenates obtained 30, 60, 120, 240 and 420 minutes after 100 mg·kg^{-1} i.p. injections. CLP257 concentrations were reported in pmol.g^{-1} (or nM) of brain weight.

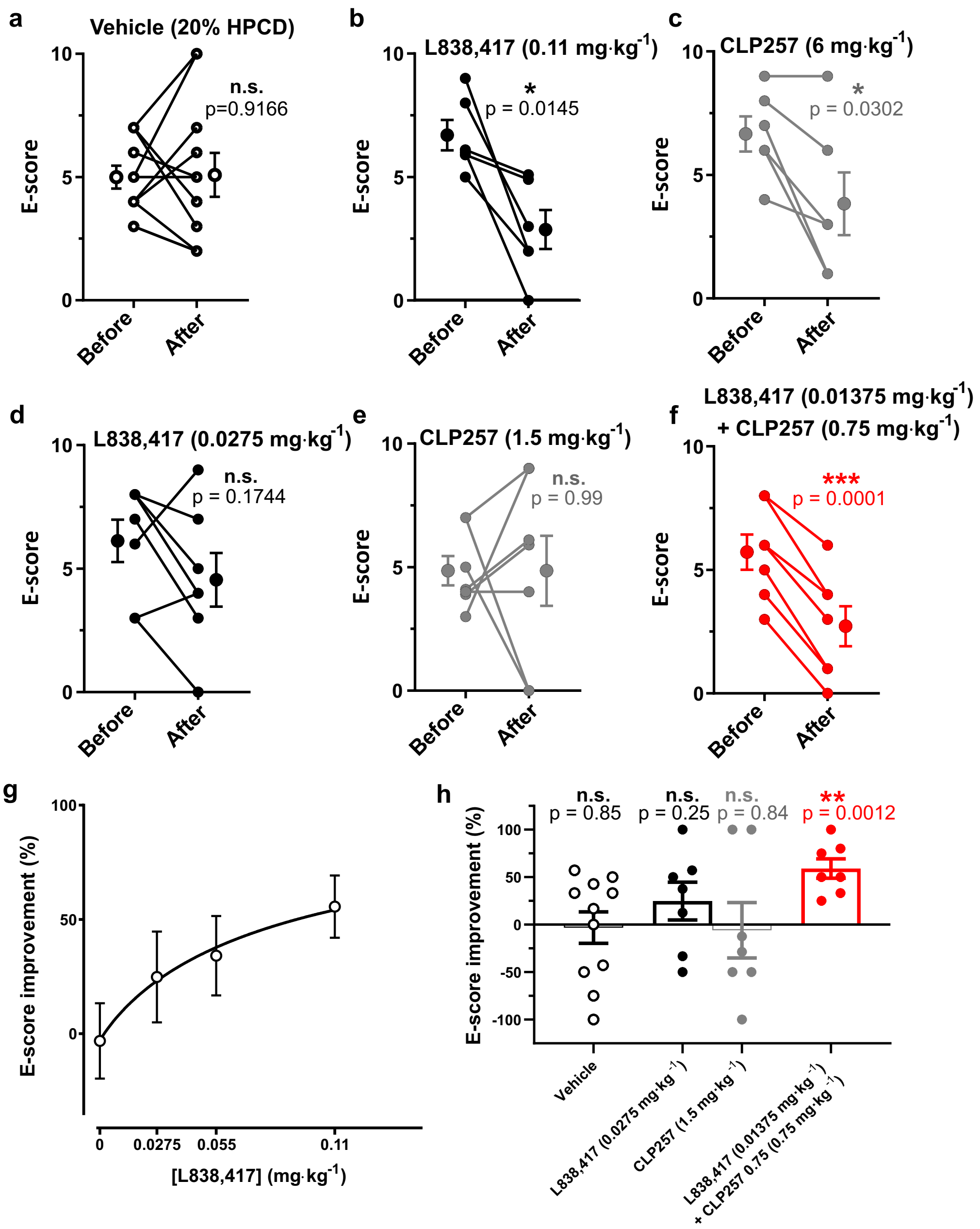

Supplementary figure 8: Complex behavioral response to liquid nitrogen application on the hindpaw confirms the synergistic action of L838,417 and CLP257.

Evoked nociceptive responses (E-score) were calculated before and after vehicle or drug injection. **a** After i.p. injection of 4 mL of vehicle (20% HPCD) no significant change in the E-score occurred. **b** I.p. injection of 4 mL of L838,417 (0.11 $mg \cdot kg^{-1}$) significantly decreased the E-score. **c** *Id.* with 6 $mg \cdot kg^{-1}$ of CLP257. **d** In contrast, a lower dose of L838,417 (0.0275 $mg \cdot kg^{-1}$) did not produce any significant analgesia. **e** Similarly, a low dose of CLP257 (1.5 $mg \cdot kg^{-1}$) did not produce significant analgesia. **f** Finally, combining both drugs to achieve a total dose equivalent to individual doses presented in c and d (i.e., L838,417 dose of 0.0275 $mg \cdot kg^{-1}$ / 2 = 0.01375 $mg \cdot kg^{-1}$ + CLP257 dose of 1.5 $mg \cdot kg^{-1}$ / 2 = 0.75 $mg \cdot kg^{-1}$) resulted in significantly reduced E-score, confirming synergistic action of the two drugs (multiple paired t-tests). **g** dose-response curve of L838,417 after liquid nitrogen application. h E-score improvements summarizing a,d-f graphics (one sample *t*-test compared to an hypothetical value of zero).

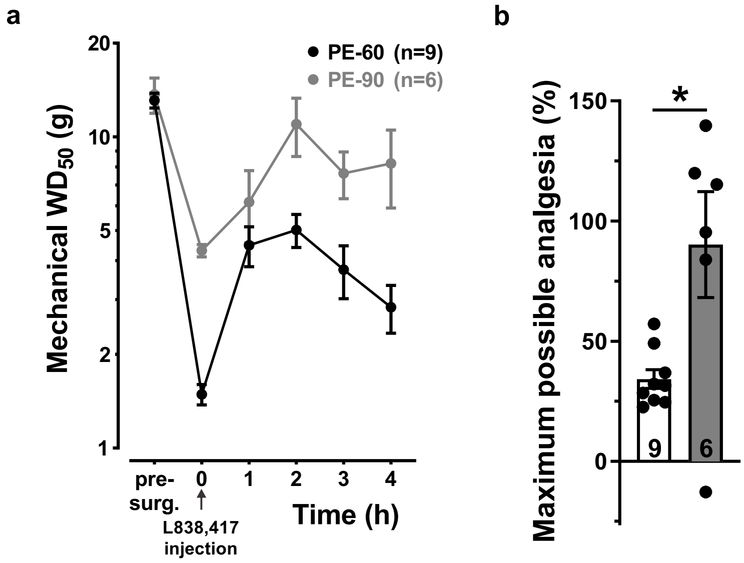

Supplementary figure 9: Peripheral nerve injury with a cuff of larger diameter.

a Mechanical Von Frey behavioral testing for 4 hours for L838,417 (3.3 mg·kg^{-1}) 14-28 days post-PNI. Results for polyethylene PE-60 (Ø = 0.76 mm) and PE-90 (Ø = 0.86 mm) cuffs (2 mm in length) were compared. Note that the withdrawal threshold decreases only to 5g when using the PE-90 tube in contrast to <2g for the PE-60 tube. **b** Maximal possible analgesia experiment using the two types of polyethylene tubes. L838,417 yields higher recovery of mechanical withdrawal threshold in the PE-90 condition than the PE-60 one. (*$P < 0.05$).

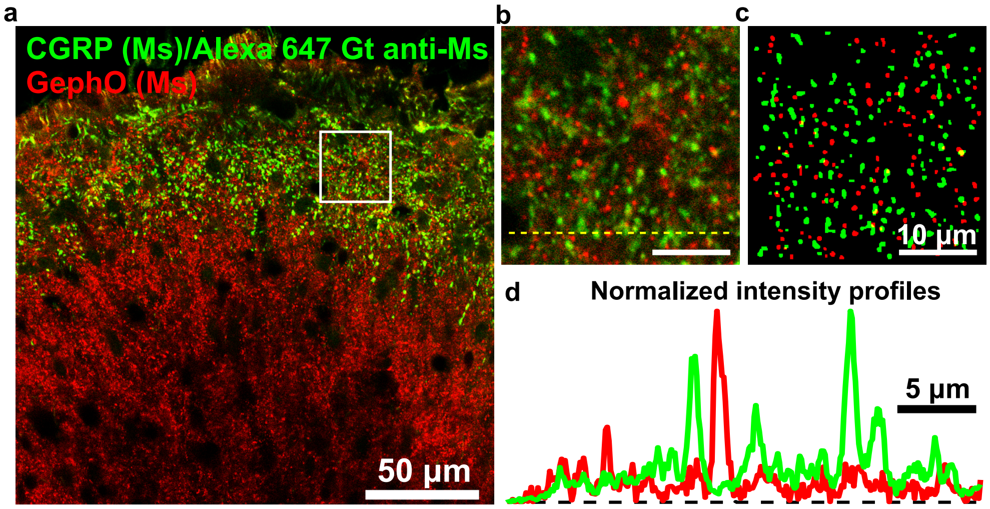

Supplementary figure 10: Reliability of the sequential co-immunostainings using multiple mouse monoclonal antibodies.

a Confocal image of the sequential co-immunostaining of an anti-CGRP mouse monoclonal primary revealed by an Alexa647 goat anti-mouse secondary antibody followed by using the fluorophore pre-coupled mAb7a anti-gephyrin-oyster primary antibody in control spinal dorsal horn (see methods). **b** Zoom-in of the white square in **a**. **c** Non-significant pixel overlap between CGRP (mouse) and gephyrin (mouse) confirming no cross-reaction of the Alexa647 goat anti-mouse secondary antibody with the pre-coupled mouse mAb7a anti-gephyrin-oyster primary antibody. **d** Normalized intensity profiles of CGRP (mouse) and gephyrin (mouse), respectively, taken from the profile line (yellow) in **b**. Ms: mouse; Gt: goat.

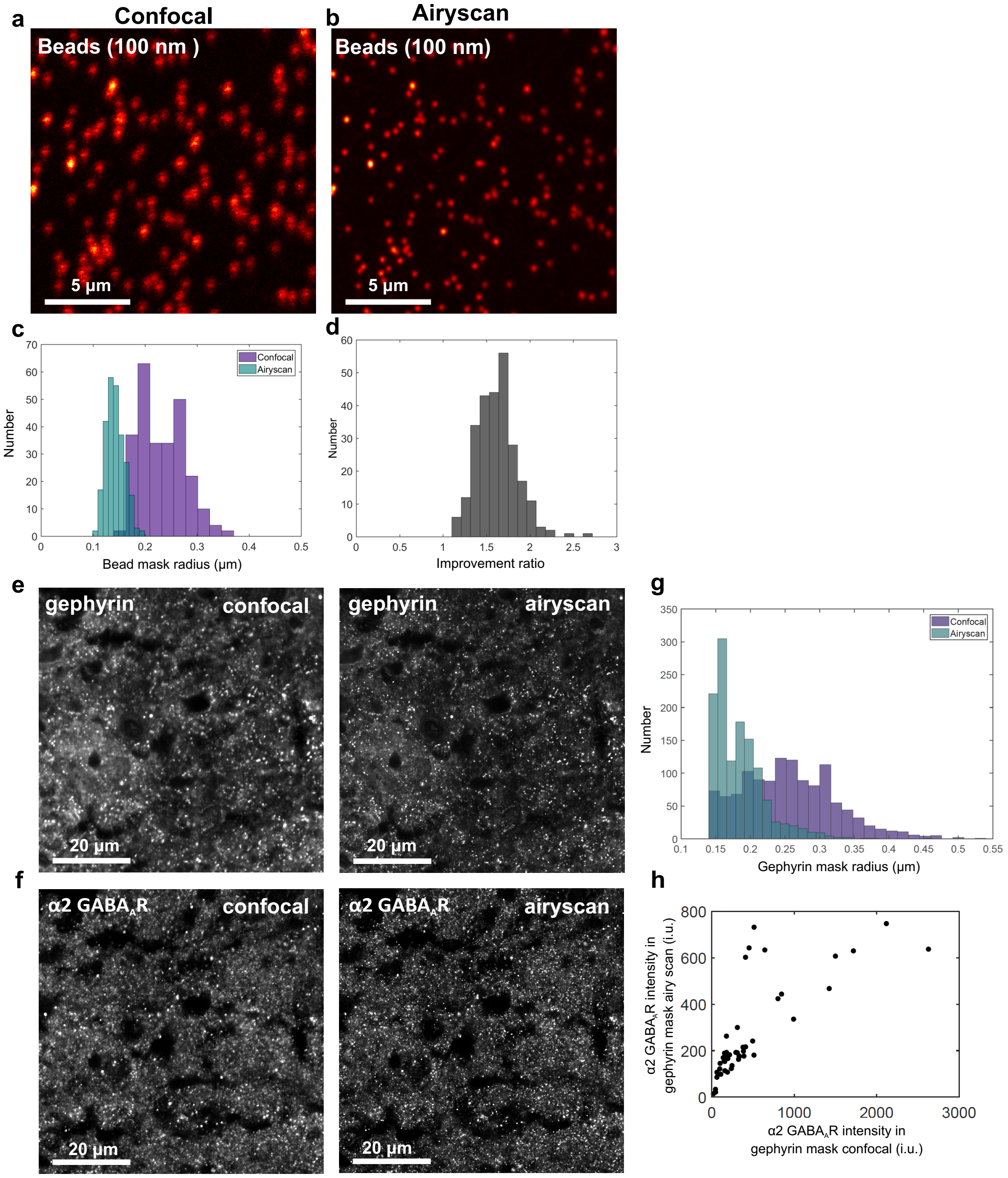

Supplementary figure 11: Comparison between confocal and multi-array detector modality.
a-b Example images of 100 nm diameter fluorescent nanospheres (Abberior TS 100 nm) revealed using a confocal and multi-array Airy scan modality. **c** Quantification of the resolution of our multi-array detector compared to confocal microscopy using 100 nm diameter fluorescent nanospheres. **d** Histogram showing the improvement between the two modalities. The full width half maximum (FWHM) of the bead intensity profiles showed a ~1.7 improvement compared to conventional confocal detection. **e-f** Gephyrin and $GABA_AR$ α2 example images revealed using both modalities. **g** Gephyrin mask area obtained using the algorithm presented in the methods section. **h** Plot comparing the intensities integrated between the two modalities.

Supplementary table 1: Real-time PCR primer sequences

Genes	Primer name	Primer sequence	Primer range	Amplimer size (bp)
Gabra1	*Gabra1*-F (5'-3')	GTCCTCTGCACTGAGAATCGC	1620-1700	81
	Gabra1-R (5'-3')	CGAATTCTTTTAAGACAGAGGCAGTA		
Gabra2	*Gabra2*-F	TCATTTTTGCTTTGTACAGTCTGACT	1475-1588	114
	Gabra2-R	GCAAGTGCAGGTCTCCTTTAGAG		
Gabra3	*Gabra3*-F	ATTTCCCGCATCATCTTCCC	1612-1696	85
	Gabra3-R	TGATAGCGGATTCCCTGTTCAC		
GPHN (Gephyrin)	*Gphn*-F	ACCTCTGGGCATGCTCTCTA	512-610	99
	Gphn-R	TGAAAGCATTCCTGAGATCC		
SLC12A5 (KCC2)	*SLC12A5-F*	GTTCCCGAAGAGACAGCTTG	2863-3046	184
	SLC12A5-R	GAGCCGCTGACTTATCCTTG		
SLC12A2 (NKCC1)	*SLC12A2 -F*	GAGGCTGACCAAAACAGAGC	2817-2982	166
	SLC12A2 -R	CCAGGGTTCAGCCATGTACT		
TFRC (Transferrin receptor)	TFRC-F	CGGCTACCTGGGCTATTGTA	246-330	85
	TFRC-R	TTCTGACTTGTCCGCCTCTT		
ACTB	*ACTB*-F	CACACTGTGCCCATCTATGA	562-833	272
	ACTB-R	CCGATAGTGATGACCTGACC		
Rpl13a	*Rpl13a-F*	GTGAGGGCATCAACATTTCT	127-368	242
	Rpl13a-R	CATCCGCTTTTTCTTGTCAT		
GAPDH	*GAPDH-F*	AGACAGCCGCATCTTCTTGT	29-235	207
	GAPDH-R	CTTGCCGTGGGTAGAGTCAT		

Primer range numbering is based on the CDS sequence.

Supplementary table 2: Primary antibodies

Antibody/Lectin	Host	Sequence	Dilution	Source	Specificity/Reference	Figure
Anti-GABA$_A$R α1	Rabbit	aa 28–43	1:1000	Sysy (#224203)	K.O. verified[1,2]	Fig. 1, 2,3
Anti-GABA$_A$R α2	Rabbit	aa 29–37	1:1000	Sysy (#224103)	K.O. verified[2,3]	Fig. 1, 2,3, Suppl. Fig. 11
Anti-GABA$_A$R α3	Rabbit	aa 29–43	1:1000	Sysy (#224303)	Sysy Western Blot	Fig. 1, 2,3
Anti-GABA$_A$R α5	Rabbit	aa 26–43	1:1000	Sysy (#224503)	K.O. verified[4]	Fig. 1, 2,3
Anti-GABA$_A$R α1	Guinea-pig	aa 01–16	1:1000	J.M. Fritschy	K.O. verified[5,6]	Suppl. Fig. 1
Anti-GABA$_A$R α2	Guinea-pig	aa 01–09	1:1000	J.M. Fritschy	K.O. verified[5,6]	Suppl. Fig. 1
Anti-GABA$_A$R α3	Guinea-pig	aa 01–15	1:1000	J.M. Fritschy	K.O. verified[7]	Suppl. Fig. 1
Anti-GABA$_A$R β3	Rabbit	aa 345-408	1:1000	W. Sieghart	Purified receptor tested[8,9]	Fig. 1, 2,3
Anti-GABA$_A$R β2,3	Mouse monoclonal	β chain	1:1000	Millipore (#MAB341)	Purified receptor tested[10,11]	Fig. 1, 2,3
Anti-GlyR α1	Mouse monoclonal	aa 01–10	1:1000	Sysy (#146111)	Sysy Western Blot[12]	Fig. 1, 2,3
Anti-Gephyrin	Mouse monoclonal	aa 264–276	1:1000	Sysy (#147011)	K.O. verified[13]	Fig. 1, 2,3, Suppl. Fig. 11
Anti-Gephyrin-Oyster	Mouse monoclonal	aa 264–276	1:1000	Sysy (#147011C3)	K.O. verified[13]	Fig. 1, 2,3, Suppl. Fig. 10
Anti-CGRP	Rabbit	aa 24–37	1:2000	Sigma (#C8198)	Peptide cross-reactivity screening[14]	Fig. 1
Anti-CGRP	Mouse monoclonal	C-terminus	1:2000	Sigma (#C7113)	Peptide cross-reactivity screening[14]	Suppl. Fig. 10
IB4-Alexa488	Synthetic	N-terminus	1:500	ThermoFisher (#I21411)	B4 cell type specific titration[15]	Fig. 1, 3, Suppl. Fig. 2,3,4
Anti-VGAT	Mouse monoclonal	aa 75-87	1:1000	Sysy (#131011)	K.O. verified[16]	Fig. 1
Anti-GAD65,67	Rabbit	aa 572-585	1:1000	Millipore (#AB-1511)	K.O. verified[17]	Fig. 1
Anti-KCC2	Rabbit	aa 932-1043	1:1000	Millipore (#07-432)	K.O. verified[18]	Fig. 3, 5, Suppl. Fig. 4

aa, amino-acid. Sysy, Synaptic System

Supplementary References

1. Wislowska-Stanek,A. *et al.* Changes in the brain expression of alpha-2 subunits of the GABA-A receptor after chronic restraint stress in low- and high-anxiety rats. *Behav. Brain Res.* **253**, 337-345 (2013).
2. Seifi,M. *et al.* Molecular and functional diversity of GABA-A receptors in the enteric nervous system of the mouse colon. *J. Neurosci.* **34**, 10361-10378 (2014).
3. Engin,E. *et al.* Neural basis of benzodiazepine reward: requirement for alpha2 containing GABAA receptors in the nucleus accumbens. *Neuropsychopharmacology* **39**, 1805-1815 (2014).
4. Perez-Sanchez,J. *et al.* alpha5GABAA Receptors Mediate Tonic Inhibition in the Spinal Cord Dorsal Horn and Contribute to the Resolution Of Hyperalgesia. *J. Neurosci. Res.* **95**, 1307-1318 (2017).
5. Fritschy,J.M., Panzanelli,P., Kralic,J.E., Vogt,K.E., & Sassoe-Pognetto,M. Differential dependence of axo-dendritic and axo-somatic GABAergic synapses on GABAA receptors containing the alpha1 subunit in Purkinje cells. *J Neurosci* **26**, 3245-3255 (2006).
6. Kralic,J.E. *et al.* Compensatory alteration of inhibitory synaptic circuits in cerebellum and thalamus of gamma-aminobutyric acid type A receptor alpha1 subunit knockout mice. *J Comp Neurol.* **495**, 408-421 (2006).
7. Fritschy,J.M. & Mohler,H. GABAA-receptor heterogeneity in the adult rat brain: differential regional and cellular distribution of seven major subunits. *J Comp Neurol.* **359**, 154-194 (1995).
8. Slany,A., Zezula,J., Tretter,V., & Sieghart,W. Rat beta 3 subunits expressed in human embryonic kidney 293 cells form high affinity [35S]t-butylbicyclophosphorothionate binding sites modulated by several allosteric ligands of gamma-aminobutyric acid type A receptors. *Mol. Pharmacol.* **48**, 385-391 (1995).
9. Kasugai,Y. *et al.* Quantitative localisation of synaptic and extrasynaptic GABAA receptor subunits on hippocampal pyramidal cells by freeze-fracture replica immunolabelling. *Eur. J. Neurosci.* **32**, 1868-1888 (2010).
10. Todd,A.J., Watt,C., Spike,R.C., & Sieghart,W. Colocalization of GABA, glycine, and their receptors at synapses in the rat spinal cord. *J. Neurosci.* **16**, 974-982 (1996).
11. Togel,M., Mossier,B., Fuchs,K., & Sieghart,W. gamma-Aminobutyric acidA receptors displaying association of gamma 3-subunits with beta 2/3 and different alpha-subunits exhibit unique pharmacological properties. *J Biol. Chem.* **269**, 12993-12998 (1994).
12. Pfeiffer,F., Graham,D., & Betz,H. Purification by affinity chromatography of the glycine receptor of rat spinal cord. *J Biol. Chem.* **257**, 9389-9393 (1982).
13. Kneussel,M. *et al.* Loss of postsynaptic GABA(A) receptor clustering in gephyrin-deficient mice. *J. Neurosci.* **19**, 9289-9297 (1999).
14. Wong,H.C. *et al.* Monoclonal antibody to rat a-CGRP: Production, characterization, and in vivo immunoneutralization activity. *Hybridoma* **12**, 93-106 (1993).
15. Murphy,L.A. & Goldstein,I.J. Five alpha-D-galactopyranosyl-binding isolectins from Bandeiraea simplicifolia seeds. *J. Biol. Chem.* **252**, 4739-4742 (1977).
16. Saito,K. *et al.* The physiological roles of vesicular GABA transporter during embryonic development: a study using knockout mice. *Mol Brain* **3**, 40 (2010).
17. Asada,H. *et al.* Mice lacking the 65 kDa isoform of glutamic acid decarboxylase (GAD65) maintain normal levels of GAD67 and GABA in their brains but are susceptible to seizures. *Biochem. Biophys. Res. Commun.* **229**, 891-895 (1996).
18. Hubner,C.A. *et al.* Disruption of KCC2 reveals an essential role of K-Cl cotransport already in early synaptic inhibition. *Neuron* **30**, 515-524 (2001).
