# Supplementary material for: Enhancing neuronal chloride extrusion rescues α2/α3 GABAA-mediated analgesia in neuropathic pain
Source: Nat Commun. 2020 Feb 13;11:869. doi: 10.1038/s41467-019-14154-6 (PMC7018745; doi:10.1038/s41467-019-14154-6)
Supplement: Supplementary file 3 — Reporting Summary [file 41467_2019_14154_MOESM3_ESM.pdf]

nature research

Corresponding author(s): DBPR / NCOMMS-18-14034B

Last updated by author(s): Nov 19, 2019

Reporting Summary

Nature Research wishes to improve the reproducibility of the work that we publish. This form provides structure for consistency and transparency in reporting. For further information on Nature Research policies, see Authors & Referees and the Editorial Policy Checklist.

Statistics

For all statistical analyses, confirm that the following items are present in the figure legend, table legend, main text, or Methods section.

n/a	Confirmed	
☐	☒	The exact sample size (n) for each experimental group/condition, given as a discrete number and unit of measurement
☐	☒	A statement on whether measurements were taken from distinct samples or whether the same sample was measured repeatedly
☐	☒	The statistical test(s) used AND whether they are one- or two-sided<br>*Only common tests should be described solely by name; describe more complex techniques in the Methods section.*
☒	☐	A description of all covariates tested
☐	☒	A description of any assumptions or corrections, such as tests of normality and adjustment for multiple comparisons
☐	☒	A full description of the statistical parameters including central tendency (e.g. means) or other basic estimates (e.g. regression coefficient) AND variation (e.g. standard deviation) or associated estimates of uncertainty (e.g. confidence intervals)
☐	☒	For null hypothesis testing, the test statistic (e.g. F, t, r) with confidence intervals, effect sizes, degrees of freedom and P value noted<br>*Give P values as exact values whenever suitable.*
☒	☐	For Bayesian analysis, information on the choice of priors and Markov chain Monte Carlo settings
☒	☐	For hierarchical and complex designs, identification of the appropriate level for tests and full reporting of outcomes
☒	☐	Estimates of effect sizes (e.g. Cohen's d, Pearson's r), indicating how they were calculated

Our web collection on statistics for biologists contains articles on many of the points above.

Software and code

Policy information about availability of computer code

Data collection	Data were collected with Excel 365 for Business.
Data analysis	Home-designed Matlab softwares or GraphPad Prism 8 were used to analyze data. All statistical tests were performed using Graphpad Prism 8.

For manuscripts utilizing custom algorithms or software that are central to the research but not yet described in published literature, software must be made available to editors/reviewers. We strongly encourage code deposition in a community repository (e.g. GitHub). See the Nature Research guidelines for submitting code & software for further information.

Data

Policy information about availability of data

All manuscripts must include a data availability statement. This statement should provide the following information, where applicable:
- Accession codes, unique identifiers, or web links for publicly available datasets
- A list of figures that have associated raw data
- A description of any restrictions on data availability

Data that are necessary to interpret, replicate and build upon the methods or findings reported in the article are available upon request.

Field-specific reporting

Please select the one below that is the best fit for your research. If you are not sure, read the appropriate sections before making your selection.

☒ Life sciences ☐ Behavioural & social sciences ☐ Ecological, evolutionary & environmental sciences

Life sciences study design

All studies must disclose on these points even when the disclosure is negative.

Sample size	No specific sample size calculations were performed. Our sample sizes were usually superior to standards in the field. They were provided in the corresponding figure legends for every experiment. Sample sizes were adequately chosen to support meaningful conclusions.
Data exclusions	No data were excluded in this study.
Replication	Most of the time, results were plotted from several replicated experiments having used even number of sham and PNI animals and identical experimental conditions.
Randomization	Animals were randomized prior to the experiment.
Blinding	For behavioral testings and analysis, experimenters were blind to the rat conditions, drug type and concentrations. For confocal image acquisition and analysis, experimenters were also blind to any conditions.

Reporting for specific materials, systems and methods

We require information from authors about some types of materials, experimental systems and methods used in many studies. Here, indicate whether each material, system or method listed is relevant to your study. If you are not sure if a list item applies to your research, read the appropriate section before selecting a response.

Materials & experimental systems

n/a	Involved in the study	
☐	☒	Antibodies
☒	☐	Eukaryotic cell lines
☒	☐	Palaeontology
☐	☒	Animals and other organisms
☒	☐	Human research participants
☒	☐	Clinical data

Methods

n/a	Involved in the study	
☒	☐	ChIP-seq
☒	☐	Flow cytometry
☒	☐	MRI-based neuroimaging

Antibodies

Antibodies used	In the supplementary table 2, a complete description of the antibodies used in this study is displayed.
Validation	When possible, the experiments were replicated using more than one type of antibodies. References validating the specificity of the antibodies used are presented in a table in the supplementary text (table 2).

Animals and other organisms

Policy information about studies involving animals; ARRIVE guidelines recommended for reporting animal research

Laboratory animals	Only adult male Sprague Dawley rats were used in this study.
Wild animals	No wild animals were used in this research.
Field-collected samples	n/a
Ethics oversight	All experimental procedures were performed in accordance with guidelines from the Canadian Council on Animal Care and the International Association for the Study of Pain.

Note that full information on the approval of the study protocol must also be provided in the manuscript.
